# Primary aortoduodenal fistula: a rare cause of massive gastrointestinal hemorrhage

**DOI:** 10.4322/acr.2021.301

**Published:** 2021-08-20

**Authors:** George St. Stoyanov, Deyan Dzhenkov, Lilyana Petkova

**Affiliations:** 1 Medical University of Varna “Prof. Dr. Paraskev Stoyanov”, Faculty of Medicine, Department of General and Clinical Pathology, Forensic Medicine and Deontology, Varna, Bulgaria

**Keywords:** Pathology, Autopsy, Aortic Aneurysm, Digestive System Fistula

## Abstract

Aortoduodenal fistula (ADF) is the most common type of aortoenteric fistula (AEF). This is a rare entity, which produces communication between an abdominal aortic aneurysm (AAA) and the gastrointestinal tract (GIT), resulting in massive gastrointestinal bleeding. AEF/ADF is difficult to recognize clinically, with the classical triad of symptoms including a pulsating, palpable mass, abdominal pain, and GIT bleeding. AEF/ADF can be classified into primary when a communication between an AAA and the GIT develops with no history of prior aortic reconstructive surgery, and secondary, where the communication is on the background of previous aortic reconstructive surgery. Herein we present a case report of a 75-year-old Caucasian male patient with a clinical history of AAA, who presented with massive GIT bleeding and expired shortly after. An autopsy revealed communication between an atherosclerotic AAA and the lower third of the duodenum.

## INTRODUCTION

Aortoenteric fistulas (AEF) are communications between an abdominal aortic aneurysm (AAA) and the gastrointestinal tract (GIT), resulting in massive intraluminal hemorrhage.^1^^–^ 3 These conditions are difficult to diagnose clinically as patients present in a severely deteriorated condition, hemodynamically unstable, with severe gastrointestinal bleeding such as hematemesis, melena, rectorrhagia, or a combination of these.^2^

Clinical history of AAA or aortic reconstructive surgery, a palpable, pulsating abdominal mass, and abdominal pain can be of aid; however, due to the rarity of the condition, the only specific findings and hence the gold standard for diagnosis remains an abdominal computer tomography (CT) scan.^2^^–^ 4

AEF can be classified based on their location, with the most common site being the lower third of the duodenum - aortoduodenal fistula (ADF), with other sites including the stomach, other parts of the small intestine, colon, and esophagus.^2^^,^ 5 ^,^ 6 Similar conditions have been described with connections to the bronchial tree (aortobronchial fistula), aorto-cardiac fistulas, and aorta-venous (aortocaval) fistulas.^6^^–^ 9 Further AEF/ADF can be classified into primary fistulas, developing de novo from direct communication between an AAA and the GIT, and secondary, where the communication between the aorta and GIT develops on the background of previous aortic reconstruction surgery.^1^

The incidence of AEF remains low, with secondary AEF occurring much more commonly, with an incidence of 0.36-1.6% of patients undergoing aortic reconstructive surgery, who can develop the condition even years after the surgery.^10^ Primary AEF is much rarer in incidence, comprising 0.04-0.07% of all autopsy cases.^1^^,^ 3 ^,^ 10 Combined yearly incidence for both primary and secondary AEF has been estimated to be 0.007 per million capita.

To date, there have been around 400 reported cases in the medical literature of AEF.^1^^,^ 2 ^,^ 11

The mortality rate of AEF is high.^1^^,^ 12 It is estimated that the mortality in untreated cases is 80-100%, with mortality in diagnosed and treated cases varying between 30% and 56% on the background of high perioperative mortality, varying from 18% to 63%.^1^^,^ 3 ^,^ 6 ^,^ 10 ^,^ 11

Herein we present a care report of a primary ADF (PADF) in a 75-year-old male patient.

## CASE REPORT

The patient, a Caucasian male, presented to an outpatient cardiology clinic, where he had been followed up. Symptoms for a visit were abdominal pain and palpitations; however, due to an episode of fainting, no electrocardiogram abnormalities, release of pelvic reservoirs, and an episode of hematemesis and melena he was referred to our institution for further diagnostic tests and treatment. At the emergency department, he presented with hematemesis and rectorrhagia, in a severely deteriorated general condition.

Vitals showed no fever, anemic mucosal surfaces, heart rate of 120 beats per minute, blood pressure of 70/50 mmHg, respiratory rate of 30 breaths per minute, with evident apneic pauses. On the background of resuscitation measures, the patient expired shortly after admission.

The whole period from the primary presentation to death was 40 minutes and from the onset of fainting, hematemesis, and melena to death - 10 minutes.

The patient was referred for autopsy due to the extremely short hospital stay and the inability for any diagnostic tests to be performed.

### Previous Medical History

**Figure 1:**
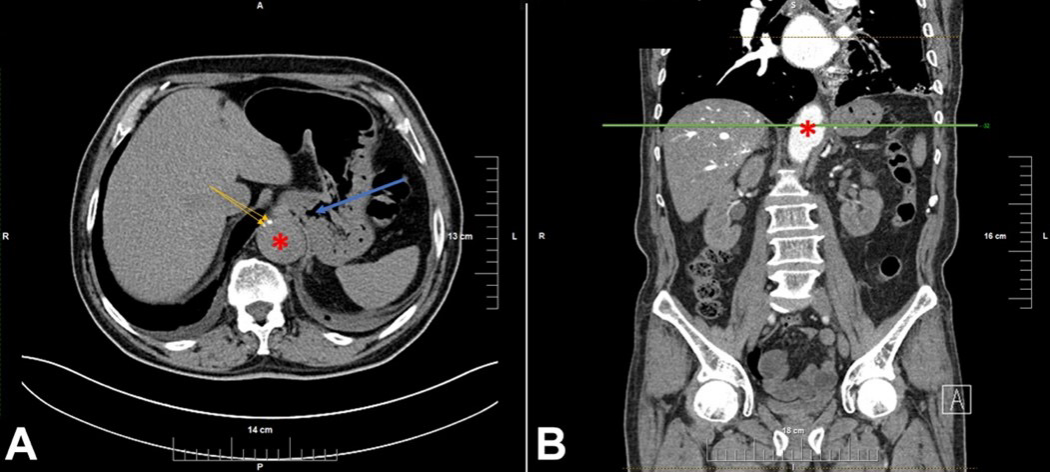
Thoracoabdominal CT scan: **A** – red asterisk indicates the abdominal aortic aneurysm, yellow arrows indicate the calcium aggregated in the atherosclerotic plaque, blue arrow points toward the duodenal lumen and the duodenal wall adjacent to the aneurysm; **B** – red asterisk indicates the lumen of the aneurysm, the green line indicates the level of section A of the figure. CT: computer tomography.

Previous medical history included pulmonary thromboembolism two months prior, when two AAA were reported on abdominal CT, both were fusiform, with the first one just below the diaphragm with a diameter of 40mm and a second one, 3mm below the left renal artery, with a diameter of 34mm (Figure 1). The patient was prescribed anticoagulant therapy, but did not follow up the treatment regimen.

Concomitant conditions included class III New York Heart Association (NYHA) heart failure, ischemic heart disease for which balloon dilatation of the left coronary artery was performed seven years’ prior, permanent atrial fibrillations, stage III hypertensive disease, with cardiac hypertrophy, bronchiectatic disease, and peripheral artery disease with subtotal occlusion of both femoral arteries.

## AUTOPSY PRESENTATION

On observation, the skin and visible mucosal surfaces were pale, the orifice of the oral cavity was stained with hematin mater, while the perineal area was stained both with hematin and clear blood. The body mass index was 21.6 (height 180cm, weight 70kg).

Section of the abdomen revealed two palpable masses in the retroperitoneal area, along the prevertebral area, the first one in the epigastric area, and the second one below the kidneys. Upper GIT was filled with hematin materials, while the lower GIT was filled with clear blood, with the mucosa being stained in some areas with hematin mater.

**Figure 2:**
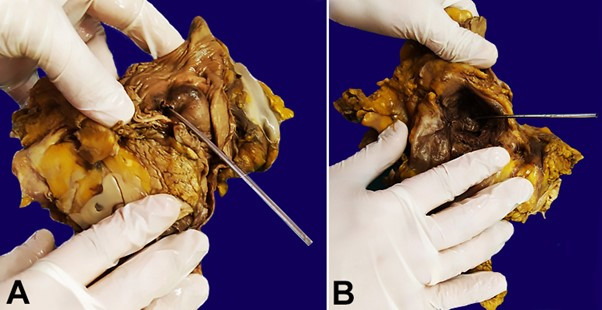
PADF post-fixation on formalin: **A** – duodenal side of the fistula (probe placed in the orifice); **B** – aortic side of the fistula (probe placed in the orifice).

**Figure 3:**
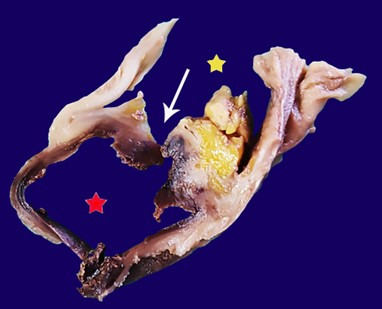
PADF post-fixation on formalin: section through the level of the fistula, the “red star” indicates the lumen of the aortic aneurysm, the “yellow star” indicates the duodenal lumen, white arrow indicates the place of the communication.

The lower third of the duodenum was adjacent to the upper mass and when opened, a communication between the duodenum and the subdiaphragmatic AAA was observed (Figure 2-3).

The internal organs showed anemic changes due to the massive hemorrhage, left-sided concentric cardiac hypertrophy (cardiac weight of 550 grams, left ventricular thickness of 25mm, right ventricular thickness of 4mm), and adrenal cortical hyperplasia were consistent with the hypertensive disease, coronary atherosclerosis was significant in the left coronary artery with occlusion of up to 60%, without thrombosis. The respiratory system showed significant pulmonary edema, and the nervous system revealed cerebral edema with cerebellar tonsillar herniation into the foramen magnum. The remaining systems did not show any gross pathological changes.

### Histological Findings

**Figure 4:**
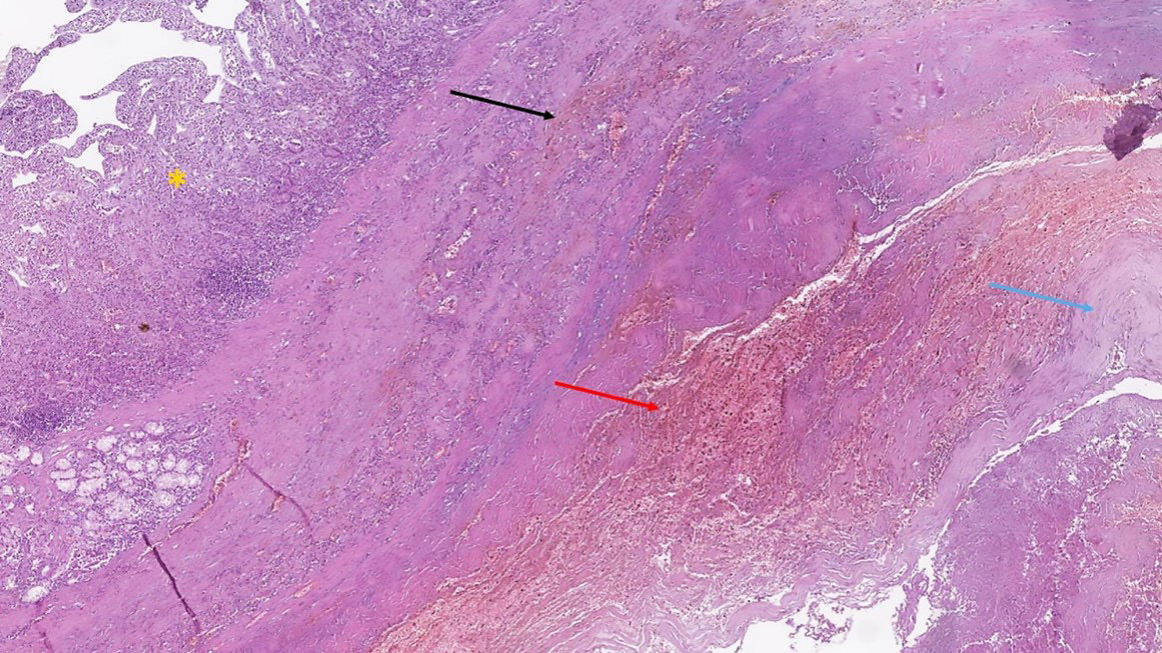
Histopathology from the area of penetration of the PADF; yellow asterisk indicates the preserved duodenal mucosa, black arrow indicates hemorrhages in the muscular layer of the duodenum, the red arrow indicates the area of penetration with diffuse hemorrhagic zones, blue arrow indicates the remnants of the aortic wall; hematoxylin and eosin stain, original magnification 40x.

Histopathology of the specimens from the area of the PADF revealed an atherosclerotic nature of the aneurysm, fibrinoid necrosis of the aortic wall, and hemorrhagic changes in the wall and mucosa of the adjacent duodenal wall, without any evidence of duodenal ulcers or ischemic change (Figure 4).

The cause of death was determined as complicated atherosclerosis of the abdominal aorta, with two atherosclerotic AAA and bilateral subtotal atherosclerotic occlusion of the femoral arteries, a PADF with massive GIT hemorrhage, and acute severe post-hemorrhagic anemia.

## DISCUSSION

It is accepted that the first description of a PADF was made by Sir Astley Paston Cooper in 1829.^2^^,^ 5 ^,^ 13 He described a patient who clinically had a pulsating tumor mass above the umbilicus, who several weeks later passed several bloody stools, fainted, and died suddenly the following day after several more episodes of bloody stools. Upon autopsy, Cooper found an AAA to which the jejunum and distal third of the duodenum had adhered. Upon opening the aneurysm, he established a connection between the AAA and the intestines 13 ^,^ 14.

AEF develop most commonly in the lower third of the duodenum, due to Its retroperitoneal location and relatively fixated nature to the other retroperitoneal structures, including the aorta. 1 ^-^ 3 ^,^ 11 Formation of AEF in other areas of the GIT Is rare due to the mobility of the small and large intestine and the need for a fixated structure for the development of the fistula, otherwise, the case presents as an AAA rupture. However, cases of AEF In other cases have been described, especially secondary AEF and in the presence of GIT adhesions. 6 ^,^ 10 ^-^ 11

In most of the cases of PADF and AEF, the AAA is of atherosclerotic origin, with other rare causes being traumatic aneurysms, mycotic aneurysms, and other rare causes such as radiation, metastasis, chronic inflammations (predominantly hematogenous disseminated tuberculosis), and diverticulitis.^5^^,^ 15

Nowadays, most cases of AEF/ADF are secondary, after aortic reconstructive surgery, with some reported cases developing years after the initial procedure.^1^^,^ 6

Clinical suspicion of the condition is scarce, due to its rarity. However, a few patients may have the clinical history of AAA, without reconstruction in the case of PADF. The clinical course is also rapid, and in most cases does not allow proper diagnostic tests to establish the reason for the GIT bleeding, such as endoscopy and CT. As seen in the literature and underlined by our case, the clinical presentation of the patients can take up to several hours; however, in fulminant presentation, death may occur within several minutes. Furthermore, classical symptoms, described as the triad of palpable, pulsating abdominal mass, abdominal pain, and GIT bleeding, may not be present in all cases, or patients may be in a deteriorated condition and unable to report them.^1^

No predicitive factors are described in the medical literature for the development of AEF/ADF, other than the presence of an AAA, or a history of aortic reconstructive surgery of the abdominal aorta, on the background of an extremely low incidence of these morbid entries.^11^

The gold standard diagnostic method remains the contrast-enhanced abdominal CT, where the defect of the aorta, as well as the presence of contrast medium leackage into the GIT, can be well visualized. 1 ^,^ 3 ^,^ 6 The treatment Is only surgical, with the repair of the AAA wall defect as well as that of the Intestinal wall, hemotransfusion for the stabilization of the patient and intensive care monitoring until discharge.^1^^,^ 3 ^,^ 11 Patient follow-up after the procedure is also highly indicated, as there is a lickelyhood for the development of a further complication, one of which Is the development of a secondary AEF.^3^^,^ 11

## CONCLUSION

As seen by the presentation of our patient and several reports from the literature AEF/PADF is a severe complication of AAA, which penetrates the GIT and results in severe internal bleeding, with an immediate threat to one's life. So far, there have been less than 400 published cases of this rare entry, described for the first time nearly 200 years ago. Clinical suspicion should be kept in mind in all patients with known aortic reconstructive surgery, as well as cases with the triad of palpable, pulsating abdominal mass, abdominal pain, and GIT bleeding. The gold standard for diagnosis is abdominal CT, with treatment being surgical intervention. Unfortunately, the mortality remains high.
